# Serum IgG N-glycans act as serum biomarkers for differentiation of cold and heat pattern in rheumatoid arthritis

**DOI:** 10.1186/s13020-025-01246-3

**Published:** 2025-11-06

**Authors:** Yilin Wang, Yu Lai, Jingrong Wang, Jiaqi Wu, Hao Yu, Yao Xiao, Liang Liu, Zishao Zhong, Hudan Pan

**Affiliations:** 1 Guangdong Provincial Hospital of Chinese Medicine, Guangdong Provincial Academy of Chinese Medical Sciences, State Key Laboratory of Traditional Chinese Medicine Syndrome, The Second Affiliated Hospital of Guangzhou University of Chinese Medicine, 111, Dade Road, Guangzhou, 510000 China; 2 Chinese Medicine Guangdong Laboratory, Zhuhai, 519000 China; 3 Department of Metabolic Diseases of Integrated Chinese and Western Medicine, The Affiliated Hospital of Guangdong Pharmaceutical University, Guangzhou, 510000 China; 4 Macau Institute for Translational Medicine and Innovation, University of Macau, Macao SAR, 999078 China

**Keywords:** Rheumatoid arthritis, Serum IgG N-glycans, TCM syndrome, Glycomics, Cold and heat patterns

## Abstract

**Background:**

To develop and validate a panel of serum IgG N-glycan biomarkers for both the diagnosis of rheumatoid arthritis (RA) and the differentiation of Traditional Chinese Medicine (TCM) syndromes in RA patients.

**Methods:**

We conducted a case–control study involving 105 patients meeting the 2010 American College of Rheumatology/European Alliance against Rheumatism RA classification criteria and 79 healthy controls. RA patients were classified according to TCM principles into cold and heat patterns. Serum IgG was enriched using titanium dioxide-porous graphitic carbon (TiO2-PGC) wafers and analyzed by high-performance liquid chromatography. IgG N-glycans were quantified using multiple reaction monitoring. Potential N-glycan biomarkers for RA diagnosis and TCM syndrome differentiation were identified and validated using multivariate data analysis.

**Results:**

Orthogonal partial least squares discriminant analysis (OPLS-DA) identified 57 N-glycans (variable importance in projection > 1) that differentiated between RA cold pattern, heat pattern, and healthy controls. Through random forest machine learning and Kruskal–Wallis testing, we identified three acidic N-glycans (5_4_0_1-a, 5_4_0_2-a, and 5_4_0_2-b) as potential diagnostic biomarkers. In the training set, receiver operating characteristic analysis demonstrated that this three-N-glycan panel effectively distinguished RA patients from healthy controls (AUC 0.90), with particularly strong discrimination between RA heat pattern and healthy controls (AUC 0.99) and between RA cold pattern and healthy controls (AUC 0.84). The robust predictive performance was further validated in an independent test set. Additionally, we developed a logistic regression model for future clinical application in predicting both RA diagnosis and its heat/cold syndrome patterns.

**Conclusion:**

This glycomics-based approach identified and validated novel N-glycan biomarkers associated with both RA diagnosis and TCM syndrome differentiation. The combination of these N-glycan biomarkers and our diagnostic model offers a promising strategy for integrating modern diagnostic techniques with TCM classification in RA management.

**Supplementary Information:**

The online version contains supplementary material available at 10.1186/s13020-025-01246-3.

## Introduction

Rheumatoid arthritis (RA) represents a complex autoimmune disorder characterized by persistent synovial inflammation, leading to irreversible bone erosion, cartilage destruction, and systemic extra-articular manifestations [1–4]. While serological markers such as rheumatoid factor (RF) and anti-citrullinated protein antibodies (ACPA) remain the cornerstone of early RA diagnosis, their clinical utility is hampered by limited sensitivity, with approximately 30% of patients presenting as seronegative. Given that early therapeutic intervention is crucial for preventing joint destruction and improving long-term outcomes, there is an urgent unmet need for novel, more sensitive biomarkers to facilitate early and accurate RA diagnosis.

Traditional Chinese Medicine (TCM) has demonstrated remarkable clinical efficacy over its millennia-long history in China [5]. Central to TCM's effectiveness is its pattern differentiation-based treatment approach, which enables personalized therapeutic strategies [6, 7]. However, the traditional TCM pattern differentiation process, heavily dependent on physicians' subjective assessment of symptoms, tongue characteristics, and pulse conditions, poses significant challenges for standardization and widespread adoption. Consequently, there is an imperative need to develop objective, technology-driven approaches for TCM pattern differentiation [8, 9]. In TCM theory, Cold and Heat patterns, representing distinct pathophysiological responses [10], account for over half of RA cases [11]. These patterns manifest as distinct symptom clusters in RA patients and necessitate different therapeutic approaches, making accurate Cold-Heat pattern differentiation crucial for both mechanistic understanding and optimal treatment selection.

Multi-omics approaches have significantly advanced biomarker discovery in RA, with significant advances through metabolomics, transcriptomics, and genomics studies [12–15]. Notably, metabolomics has enabled early RA diagnosis with promising accuracy (93% sensitivity, 70% specificity) using a 52-metabolite panel [16]. This approach has further revealed distinct metabolic signatures between RA Cold and Heat patterns, primarily in amino acid, carbohydrate, and lipid metabolism. While Cold pattern exhibits enhanced fat and protein mobilization, Heat pattern shows increased oxidative stress and bone destruction [17–19].

Glycomics, an emerging field focusing on glycan structure and function in biological systems, has revealed critical insights into autoimmune diseases through serum IgG analysis [20–23]. Notably, changes in antibody glycosylation precede arthritis development and correlate with disease activity and inflammation. N-linked glycans have emerged as objective markers for RA diagnosis [24–27]. As protein glycosylation reflects both genetic and environmental influences on protein function, it aligns well with TCM syndrome differentiation, which characterizes pathological manifestations resulting from internal–external environmental interactions. While our previous work identified two acidic N-glycans as potential biomarkers for seronegative RA [28], the glycomic signatures distinguishing RA Cold and Heat patterns remained unexplored. In this study, we conducted comprehensive analysis of serum IgG glycosylation patterns in RA patients and healthy controls (HCs), successfully identifying three N-glycan biomarkers that effectively differentiate between RA Cold and Heat patterns.

## Materials and methods

### Patients

A total of 105 patients were recruited from the rheumatology departments of the First Affiliated Hospital of Tianjin University of Chinese Medicine and the First Affiliated Hospital of Hunan University of Chinese Medicine from April 2018 to July 2020. These patients were diagnosed with RA according to the 2010 American College of Rheumatology (ACR)/European Alliance against Rheumatism (EULAR) classification criteria [29]. Additionally, 79 healthy volunteers, whose age and gender were matched with the RA patients, were enrolled from the physical examination centers of the First Affiliated Hospital of Hunan University of Chinese Medicine and Zhuhai Hospital of Integrated Traditional Chinese and Western Medicine. Based on TCM syndrome differentiation, all RA patients were classified into two distinct patterns: cold-dampness impeding syndrome and dampness-heat impeding syndrome. The study protocol (No. 20201215035) was approved by the institutional ethics committee of Zhuhai Hospital of Integrated Traditional Chinese and Western Medicine, and all participants provided informed consent.

### Inclusion criteria and exclusion criteria

Inclusion criteria were defined as follows: patients aged 18 years or older meeting the 2010 ACR/EULAR diagnostic criteria for RA, and those diagnosed with either cold-dampness impeding syndrome or dampness-heat impeding syndrome. Exclusion criteria included a history of severe cardiovascular, liver, kidney, mental, or hematological diseases, as well as those with severe infections, organ failure, malignant tumors, or pregnancy. Patients diagnosed with other TCM syndrome types were also excluded from the study.

### TCM pattern differentiation and quality control

To ensure diagnostic precision and mitigate the inherent subjectivity in TCM, the TCM syndrome classification in this study was strictly based on the Guideline for Diagnosis and Treatment of Rheumatoid Arthritis based on TCM Syndromes (T/CACM 013–2017), issued by the China Association of Chinese Medicine (Supplementary Table 1). We focused on two clinically dichotomous patterns: the cold-dampness impeding syndrome (cold pattern) and the dampness-heat impeding syndrome (heat pattern). A diagnosis of the cold pattern was confirmed by the presence of its primary symptoms—joint pain that is cold to the touch without local redness, which is characteristically aggravated by cold exposure and alleviated by warmth. Conversely, the heat pattern was identified by its hallmark symptoms of swollen, hot, painful joints that feel warm or hot upon palpation. For a definitive classification, a patient had to present either two primary symptoms, or one primary and two secondary symptoms, corroborated by corresponding tongue and pulse findings, thereby establishing a clear and reproducible diagnostic framework.

To ensure diagnostic reliability and reproducibility, a rigorous quality control procedure was implemented. All investigators participated in centralized training sessions to standardize terminology and diagnostic procedures. Each of the 105 RA patients was independently evaluated by two senior TCM physicians (each with the title of associate chief physician or chief physician). Their diagnosis was based on a comprehensive evaluation of data from patient-reported symptom questionnaire, tongue appearance, and pulse conditions. This robust process yielded a high degree of inter-rater reliability, with an initial diagnostic consensus reached in 103 of the 105 cases (98.1%). For the two remaining cases with initial disagreement, a diagnostic panel was formed including a third, more senior chief physician, and a final consensus diagnosis was reached by majority rule. This multi-faceted, consensus-based protocol was designed to ensure the validity and consistency of the TCM pattern classification, which is crucial for the subsequent biomarker analysis.

### Human serum collection

A total of 105 serum samples from RA patients (41 cases of cold-dampness impeding syndrome and 64 cases of dampness-heat impeding syndrome) and 79 serum samples from healthy individuals were collected. Serum samples (5 mL) were obtained from fasting venous blood before breakfast. After centrifugation and extraction, the upper serum layer was stored at − 80 °C for subsequent glycomics analysis.

### Detection of N-glycan abundance from serum

Serum N-glycan analysis was performed as previously described [28] using high-performance liquid chromatography (HPLC). Briefly, IgG was purified from serum using protein A. The purified IgG was then diluted, incubated, and washed with distilled water to remove other proteins. The eluate was collected, dried under high-speed vacuum, and stored at − 80 °C. Finally, N-glycan samples were quantitatively profiled using the multiple reaction monitoring (MRM) method. Data were processed using Agilent MassHunter B.06.00 software, and the abundance of N-glycans was quantified based on the peak area of the MRM chromatogram. The total N-glycans for each IgG sample were expressed as a percentage of the sum of the peak areas.

### Bioinformatics analysis

Raw data were imported into R (version 4.0.5) for normalization and multivariate statistical analysis. Principal component analysis (PCA) and orthogonal partial least squares discriminant analysis (OPLS-DA) were employed to explore the data (SIMCA version 14.1, Umetrics, Sweden). Hierarchical clustering of N-glycans was conducted using the ‘pheatmap’ package, while PCA maps, differential N-glycan spectra, scatter plots, and violin plots were generated using the ‘ggplot2’ package in R (version 4.0.5).

### Statistical analysis

The statistical significance of N-glycan data was calculated using R (version 4.0.5). Quantitative data were presented as mean ± standard deviation (SD), and count data were presented as percentages (n %). T-tests or nonparametric tests were used to compare quantitative variables. For multi-group comparisons, Kruskal–Wallis tests were performed, and p-values from subsequent pairwise post-hoc tests were adjusted using the Bonferroni correction to account for multiple comparisons. A two-sided P-value < 0.05 was considered statistically significant. Receiver operating characteristic (ROC) curves were generated using R to evaluate the diagnostic performance.

A logistic regression model was developed to predict both RA diagnosis and its TCM syndrome patterns. The model was trained on the dataset from the First Affiliated Hospital of Hunan University of Chinese Medicine (training set) and validated on the dataset from the First Affiliated Hospital of Tianjin University of Chinese Medicine (test set).

## Results

### Participant characteristics

A total of 105 RA patients (41 cases of cold-dampness impeding syndrome type, 64 cases of dampness-heat impeding syndrome type) and 79 healthy individuals were enrolled in our study. The average ages of the RA patients were 54.43 years while that of healthy participants were 54.52 years. Clinical characteristics and baseline demographic of RA patients with different TCM syndromes are shown in Table 1. The baseline of RA cold and heat pattern groups were generally similar. There were no significant differences in any of the following metrics (*P* > 0.05).

**Table 1:** Clinical data at baseline in RA patients with different TCM syndromes

Characteristics	CDIS (n = 41)	DHIS (n = 64)
Age (SD), years	54.46 (6.28)	54.67 (6.86)
Female, n (%)	37 (90.24%)	57 (89.06%)
Disease duration (SD), years	6.94 (7.16)	7.09 (7.53)
ESR (SD), mm/h	50.54 (35.13)	55.02 (30.86)
CRP (SD), mg/l	32.66 (33.62)	34.07 (42.99)
RF (SD), U/ml	217.59 (315.32)	249.75 (388.42)
Anti-CCP, positive rate	34/41 (82.93)	55/64 (85.94)
28TJC	7.61 (3.87)	7.84 (5.41)
28SJC	8.78 (4.58)	9.20 (4.93)
HAQ-DI	1.13 (0.59)	1.18 (0.52)
Pain (mm)	33.66 (9.75)	35.16 (12.47)
PGA (mm)	48.66 (11.67)	51.25 (15.51)
PhGA (mm)	46.71 (13.07)	48.59 (15.24)
DAS28 (CRP)	4.98 (1.48)	5.00 (1.63)
DAS28 (ESR)	5.52 (1.21)	5.71 (1.21)

CDIS: Cold-dampness impeding syndrome; DHIS: Dampness-heat impeding syndrome. 28TJC: The tender count of 28 joints; 28SJC: The swollen count of 28 joints. DAS: Disease Activity Score

### Detection of significant N-glycans from raw data

We determined the N-glycan profiling in serum samples from RA patients and HCs. A total of 172 N-glycans were detected in all samples. As an initial screening step in our multi-stage biomarker identification strategy, OPLS-DA was employed. This identified 57 N-glycans with a variable importance in projection (VIP) value > 1 that contributed to the differentiation between RA Cold pattern, RA Heat pattern, and HCs. These N-glycans, which included 37 neutral and 20 acidic N-glycans (Supplementary Table 2), were considered for further selection. Differential N-glycans for pairwise comparisons (RA Cold vs. HCs, RA Heat vs. HCs, RA Cold vs. RA Heat) were also identified (Supplementary Table 3).

**Fig. 1:**
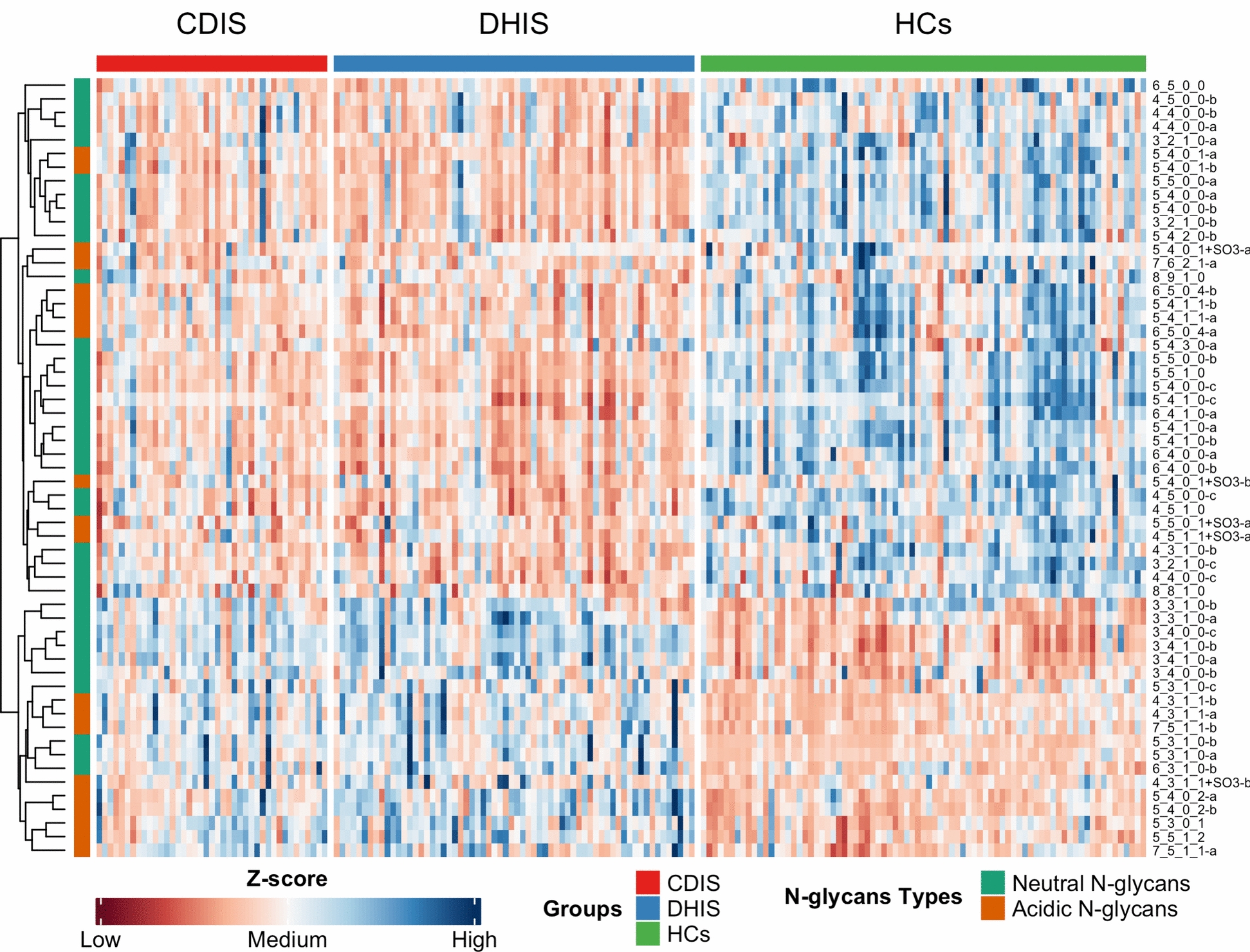
Hierarchical cluster analysis of 57 N-glycans differentiating three groups. Red color represents expression quantity up-regulation while blue color represents expression quantity down-regulation. 41 RA cold samples cluster on the left, 64 RA heat samples cluster in the middle, while the 79 healthy control samples cluster on the right

Moreover, the PCA maps were drawn with the screened N-glycans based on VIP value > 1. PCA results show that three groups of RA Cold, RA Heat and HCs were segregated (Supplementary Fig. 1). Furthermore, we conducted hierarchical cluster analysis of the expression quantity of 57 N-glycans differentiating three groups. As shown in Fig. 1, there were different levels of N-glycan expression between cold-dampness impeding syndrome group and dampness-heat impeding syndrome group, suggesting differences between cold and heat pattern of RA. Moreover, the RA cold group and RA heat group were clustered separately from healthy control group, indicating that there were significant N-glycan differences between RA patients and healthy individuals (Fig. 1).

### Differential N-glycan spectrum

HPLC analysis revealed distinct N-glycan signatures among patient groups and healthy controls. OPLS-DA identified 28 differentially expressed N-glycans (VIP > 1.5). Quantitative analysis revealed 16 significantly upregulated N-glycans (3_2_1_0-b, 3_2_1_0-c, 3_4_1_0-a, 3_4_1_0-b, 5_4_0_1 + SO3-a, 5_4_0_0-a, 5_4_0_0-c, 5_4_0_1-a, 5_5_0_0-a, 5_5_0_0-b, 5_5_1_0, 6_4_0_0-a, 6_4_0_0-b, 6_4_1_0-a, 6_5_0_4-a, 6_5_0_4-b) in RA patients (*P* < 0.05), while the remaining N-glycans were significantly downregulated (*P* < 0.05) compared to healthy controls. These altered N-glycan profiles distinguished both DHIS and CDIS RA subtypes from healthy controls, indicating syndrome-specific N-glycan signatures in RA.

### Identification of potential diagnostic N-glycan biomarkers for RA cold pattern, heat pattern and healthy controls

To identify a minimal yet robust panel from the 57 initial candidates, we implemented a systematic "funnel-down" feature selection strategy. First, to reduce the feature set and mitigate the risk of overfitting, a recursive feature elimination (RFE) method based on a random forest algorithm was applied. This process iteratively modeled the data and removed the least important features, narrowing the list down to the 10 most consistently influential N-glycans: “5_5_1_0”, “5_4_0_0-c”, “5_5_0_0-b”, “5_4_0_1-a”, “5_4_0_2-a”, “5_4_0_2-b”, “5_4_0_0-a”, “5_3_1_0-b”, “4_5_1_0”, and “3_2_1_0-b”. Among these top 10 candidates, “5_4_0_1-a”, “5_4_0_2-a”, and “5_4_0_2-b” are acidic glycans, while the remainder are neutral.

**Fig. 2:**
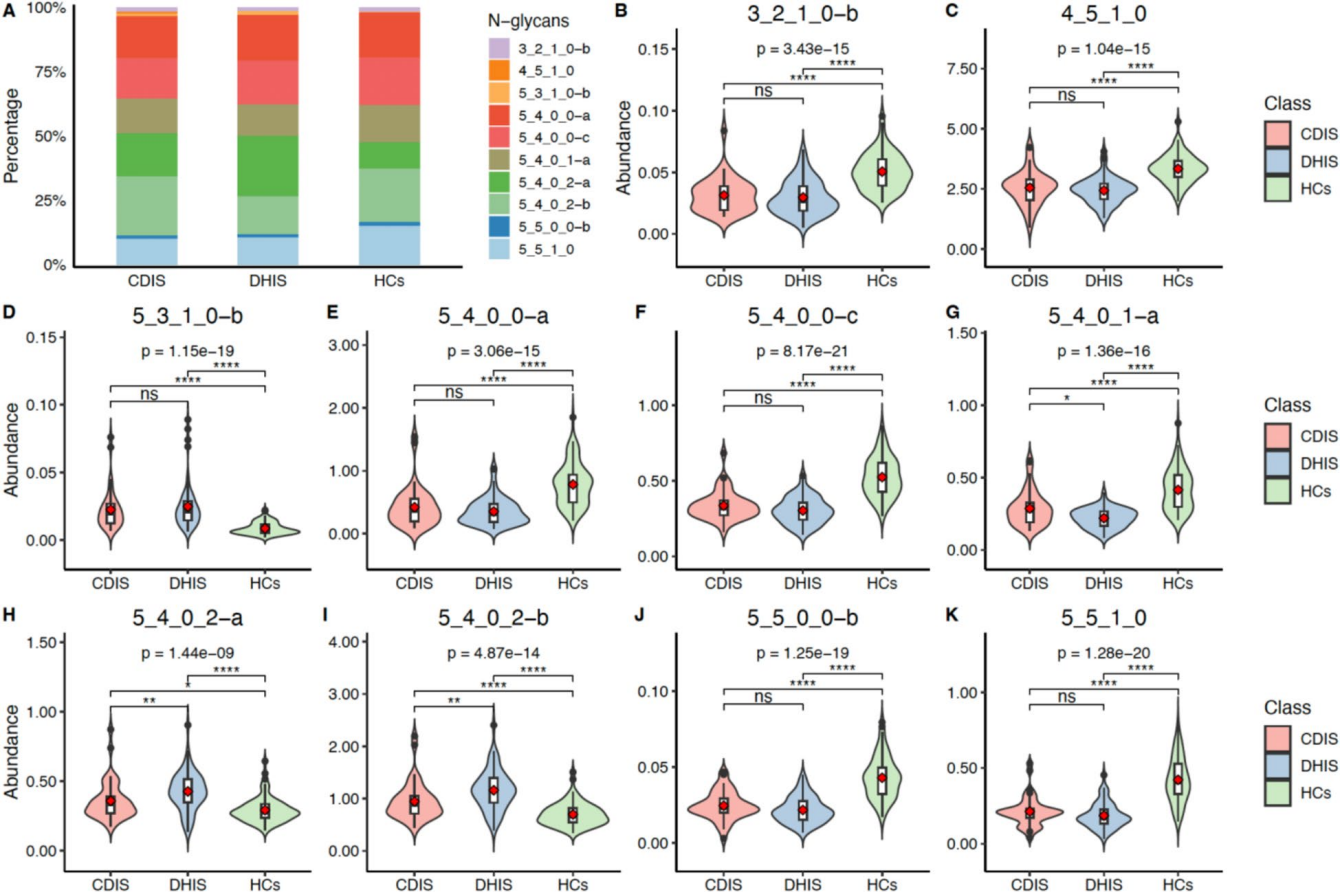
The percentage content and relative abundance of the 10 differential N-glycans in three groups. **A** The comparison of percentage content of 10 N-glycans in CDIS, DHIS, HCs groups. The y axis corresponds to the percentage of N-glycan expression content, while the x axis represents the three groups. **B**–**K** The combination of scatter plot with violin plot graphs show the serum abundances of 10 N-glycans. CDIS: Cold-dampness impeding syndrome; DHIS: Dampness-heat impeding syndrome; HCs: Healthy Controls. *P < 0.05, **P < 0.01, ***P < 0.001

In the final selection step, our goal was to identify the most parsimonious panel with the most comprehensive discriminatory power. To achieve this, we first applied the Kruskal–Wallis test to the 10 candidate N-glycans, followed by post-hoc pairwise comparisons with Bonferroni correction to control for multiple testing. While other glycans showed some level of differentiation, the three acidic N-glycans—“5_4_0_1-a”, “5_4_0_2-a”, and “5_4_0_2-b”—uniquely demonstrated statistically significant differences across the pairwise comparisons between the RA Cold, RA Heat, and Healthy Control groups (adjusted *P* < 0.05). This indicated that these glycans could not only distinguish RA patients from healthy individuals but also differentiate between the cold and heat patterns. The relative content of all 10 candidates is shown in Fig. 2A, while their individual quantified abundances are detailed in Fig. 2B–K, visually confirming the robust discriminatory power of the three selected biomarkers. The relative abundance of all 10 N-glycans is provided in Supplementary Table 4.

### Verification of biomarkers by ROC curves

**Fig. 3:**
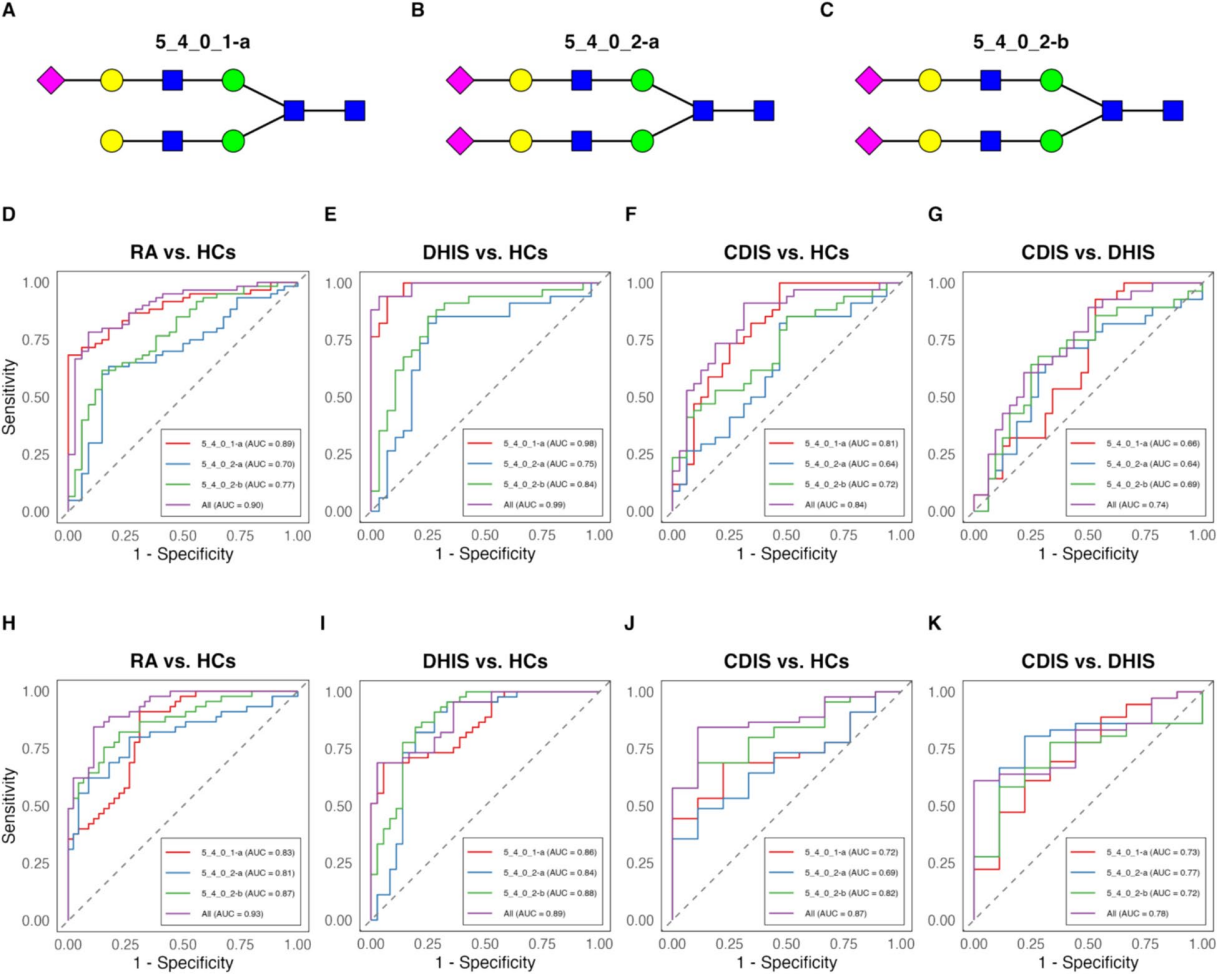
Performance evaluation of three predictive N-glycan biomarkers for RA classification and subtype differentiation. **A**–**C** Structural representations of the three identified N-glycan biomarkers 5_4_0_1-a, 5_4_0_2-a, and 5_4_0_2-b. **D**–**G** ROC curves from the training set showing diagnostic performance for: **D** RA versus HCs, **E** DHIS versus HCs, **F** CDIS versus HCs, and **G** CDIS versus DHIS. **H**–**K** ROC curves from the independent testing set validating diagnostic performance for: **H** RA versus HCs, **I** DHIS versus HCs, **J** CDIS versus HCs, and **K** CDIS versus DHIS. Each plot shows individual performance of the three N-glycans and their combined predictive power. Analyses were performed using R Studio V.4.0.5. CDIS: Cold-dampness impeding syndrome; DHIS: Dampness-heat impeding syndrome; HCs: Healthy Controls

According to the last step, we identified three acidic N-glycans (5_4_0_1-a, 5_4_0_2-a, and 5_4_0_2-b) as potential biomarkers for RA diagnosis and pattern classification (Fig. 3A–C), and evaluated using ROC curve analysis in both training and validation sets. These glycomic biomarkers demonstrated robust discriminatory power, particularly in the training set for RA vs. HCs (AUC 0.90, Fig. 3D), and was further validated in the testing set (AUC 0.93, Fig. 3H). The biomarkers showed exceptional performance in distinguishing DHIS from HCs (AUC 0.99 for training set, and AUC 0.89 for testing set, Fig. 3E, I). For CDIS vs. HCs, the markers maintained good diagnostic accuracy (AUC 0.84 for training set, and AUC 0.87 for testing set, Fig. 3F, J). Notably, these biomarkers could effectively differentiate between DHIS and CDIS subtypes (AUC 0.74 for training set, and AUC 0.78 for testing set, Fig. 3G, K). The consistent performance across both training and testing sets validates these glycomic signatures as robust biomarkers for both RA diagnosis and syndrome differentiation, offering potential clinical utility in patient stratification and personalized treatment approaches.

### Logistic regression model predicting TCM syndrome pattern of RA

Using the identified N-glycan biomarkers, we developed a multivariate logistic regression model to predict RA patterns. The model incorporates three N-glycans (5_4_0_1-a, 5_4_0_2-a, and 5_4_0_2-b) as predictor variables and generates probability scores for RA, DHIS, and CDIS status. The derived probability equations are:

$$\text{Pr}(\text{RA})=\frac{1}{1+\text{ exp}(-1.516+9.431\times (5\_4\_0\_1-\text{a}) + 9.489\times (5\_4\_0\_2-\text{a}) - 6.291\times (5\_4\_0\_2-\text{b}))}$$

$$\text{Pr}(\text{DHIS})=\frac{1}{1+\text{ exp}(23.520-96.514\times (5\_4\_0\_1-\text{a}) -11.995\times (5\_4\_0\_2-\text{a}) +8.531\times (5\_4\_0\_2-\text{b}))}$$

$$\text{Pr}(\text{CDIS})=\frac{1}{1+\text{ exp}(0.7553-7.2356\times (5\_4\_0\_1-\text{a}) -8.8364\times (5\_4\_0\_2-\text{a}) +5.6855\times (5\_4\_0\_2-\text{b}))}$$

where the probability values represent the likelihood of an individual being classified into each respective category based on their N-glycan expression levels.

## Discussion

The typical characteristic of RA is chronic inflammation which leads to irreversible joint damage and significant disability. Treatment based on TCM syndrome differentiation can effectively prevent joint damage and slow down the progression of RA. IgG antibodies act as proinflammatory mediators of the humoral immune response [30–32]. As a post-translational modification, N-glycosylation affects interactions between antibodies and receptors. The altered antibody glycosylation is related to the pathogenesis of RA [33–35]. It has been verified that altered antibody glycosylation has generated before the onset of arthritis and had positive relation to disease activity and inflammation level [36, 37]. Glycosylation may lead to pathologic modifications and ultimately promote the development of RA.

The core components of these glycan structures include N-acetylglucosamine and mannose residues, which can be further modified by terminal galactose or sialic acid residues [38]. The inflammatory functions of IgG antibodies are closely associated with glycosylation in their fragment crystallizable (Fc) region [23, 39]. In patients with RA, reduced galactosylation of IgG (specifically G1 and G2 forms) and increased levels of agalactosylated G0F glycoforms have been observed, stemming from the absence of terminal galactose (Gal) or sialic acid residues in the Fc region [27, 40]. These agalactosylated IgG (IgG0) molecules can trigger autoantibody production through immune recognition. Moreover, the absence of terminal galactose facilitates interactions between exposed GlcNAc residues and mannose-binding protein (MBP) in vivo, subsequently activating complement cascades and promoting inflammation [41]. Thus, antibody glycosylation functions as a molecular switch that can convert antibodies to an autoreactive state, potentially initiating autoimmune responses. The degree of IgG galactosylation correlates with RA disease progression, suggesting that IgG N-glycans could serve as promising biomarkers for RA diagnosis.

In this study, we employed a combined approach using HPLC and TiO2-PGC chip technology to quantitatively profile N-glycans from RA patients and healthy volunteers. Through comprehensive N-glycan data analysis using OPLS-DA, we identified 57 N-glycans (VIP value > 1) that could distinguish between RA cold pattern, heat pattern, and healthy individuals. Both PCA mapping and hierarchical cluster analysis revealed distinct clustering among these three groups. Further refinement using a more stringent criterion (VIP value > 1.5) yielded 28 N-glycans, which were used to generate a differential N-glycan profile. The resulting profile, characterized by distinct peaks and troughs, clearly demonstrated variations in N-glycan patterns associated with both disease status and TCM pattern differentiation of RA. These findings highlight substantial differences in N-glycan profiles among healthy controls and RA patients with cold and heat patterns.

Further analysis using random forest algorithms identified 10 representative N-glycans as potential diagnostic biomarkers. Among these, three N-glycans (5_4_0_1-a, 5_4_0_2-a, and 5_4_0_2-b) showed statistically significant differences (*P* < 0.05) in relative serum abundance among healthy controls and RA patients with cold and heat patterns, as determined by two-sample T-tests. ROC curve analysis revealed that the combination of these three N-glycans demonstrated robust discriminatory power, with AUC values of 0.959 for distinguishing dampness-heat impeding syndrome from healthy individuals, and 0.841 for differentiating cold-dampness impeding syndrome from healthy controls. Additionally, these three N-glycan biomarkers were incorporated into a logistic regression model to predict RA heat and cold syndrome patterns.

Notably, the behavior of the three identified acidic N-glycans reveals a more complex picture of sialylation in RA. While previous studies have demonstrated that reduced N-glycan sialylation correlates with increased pro-inflammatory and pathogenic effects in RA [24, 42], with RA patients exhibiting lower IgG sialylation levels compared to healthy individuals [25, 43, 44], a pathogenic role further supported by evidence from collagen-induced arthritis (CIA) mouse models where sialylation loss exacerbated joint inflammation [42], our findings present a paradoxical pattern. Specifically, we observed in RA patients that the monosialylated glycan 5_4_0_1-a was decreased, while the disialylated glycans 5_4_0_2-a and 5_4_0_2-b were significantly elevated. This suggests the impact in RA is not a simple monolithic decrease but a profound dysregulation of glycosylation homeostasis. This dysregulation likely stems from the altered activity of specific sialyltransferases, the enzymes responsible for attaching sialic acids to glycan chains [45].

This dysregulation may correspond to molecular-level differences that distinguish the 'cold' and 'heat' patterns. The 'heat' pattern, clinically representing a more intense inflammatory state with symptoms like redness and warmth, showed the most extreme changes—the greatest decrease in 5_4_0_1-a and the highest increase in the disialylated structures. The more intense inflammatory milieu of the 'heat' pattern could lead to more profound alterations in these enzymatic pathways compared to the 'cold' pattern. This distinction is of high biological significance, as the biological function of sialylated glycans is highly dependent on their specific linkage (e.g., α2,3- vs. α2,6-), which dictates their interaction with immune receptors [46]. The differential enzymatic activity could plausibly explain our paradoxical observation: a decreased synthesis of certain monosialylated structures alongside an increased production of specific disialylated forms. Thus, the unique N-glycan signatures may not only be biomarkers but also reflect the specific pathophysiology of each TCM syndrome.

In summary, our integrative analysis of serum N-glycan abundance data successfully identified differential N-glycan profiles that clearly distinguished among RA cold pattern, heat pattern, and healthy individuals. We characterized diverse N-glycans associated with both RA diagnosis and TCM pattern differentiation. The identification of specific N-glycan combinations as biomarkers and the development of a diagnostic model provides a potential approach for discriminating between cold and heat subtypes of RA. However, our study has two main limitations. First, these N-glycan biomarkers await validation in prospective cohorts. Second, the relationship between these biomarkers and RA severity remains unexplored. Future studies with expanded patient cohorts will be necessary to validate the clinical utility of these biomarker combinations and the established logistic regression model for RA diagnosis and TCM syndrome differentiation. Additionally, we plan to investigate the dynamic changes in total serum and IgG glycosylation patterns across different phases of RA to identify potential prognostic markers.

## Conclusion

In this study, we employed an integrated glycomics approach combining HPLC and TiO2-PGC chip technology to characterize N-glycan profiles in RA and its TCM subtypes. We identified distinct N-glycan signatures that differentiate not only between RA patients and healthy individuals but also between cold and heat TCM subtypes of RA. The combination of specific N-glycan biomarkers and our N-glycan-based diagnostic model presents novel approaches for both RA diagnosis and TCM pattern differentiation. These methods may facilitate patient stratification and inform therapeutic decision-making in RA management.

## Supplementary Information

Supplementary Material 1

Supplementary Material 2

Supplementary Material 3

Supplementary Material 4

Supplementary Material 5

Supplementary Material 6

## Data Availability

The data supporting this study's findings are available from the originating institutions ([First Affiliated Hospital of Tianjin University of Chinese Medicine, First Affiliated Hospital of Hunan University of Chinese Medicine, Zhuhai Hospital of Integrated Traditional Chinese and Western Medicine]) but are not publicly available due to licensing restrictions. They can be obtained from the corresponding author upon reasonable request and pending institutional permission.

## Abbreviations

RA: Rheumatoid arthritis
TCM: Traditional Chinese medicine
CDIS: Cold-dampness impeding syndrome
DHIS: Dampness-heat impeding syndrome
HCs: Healthy controls
CRP: C-reactive protein
ESR: Erythrocyte sedimentation rate
RF: Rheumatoid factor
HPLC: High performance liquid chromatography
MRM: Multiple reaction monitoring
PCA: Principal component analysis
OPLS-DA: Orthogonal partial least squares-discriminant analysis
VIP: Variable importance in the projection
SD: Standard deviation
ROC: Receiver operating characteristic
AUC: Area under the curve
CIA: Collagen-induced arthritis
