# Supplementary material for: Serum IgG N-glycans act as serum biomarkers for differentiation of cold and heat pattern in rheumatoid arthritis
Source: Chin Med. 2025 Nov 6;20:184. doi: 10.1186/s13020-025-01246-3 (PMC12590853; doi:10.1186/s13020-025-01246-3)
Supplement: Supplementary file 1 — Supplementary Material 1 [file 13020_2025_1246_MOESM1_ESM.doc]

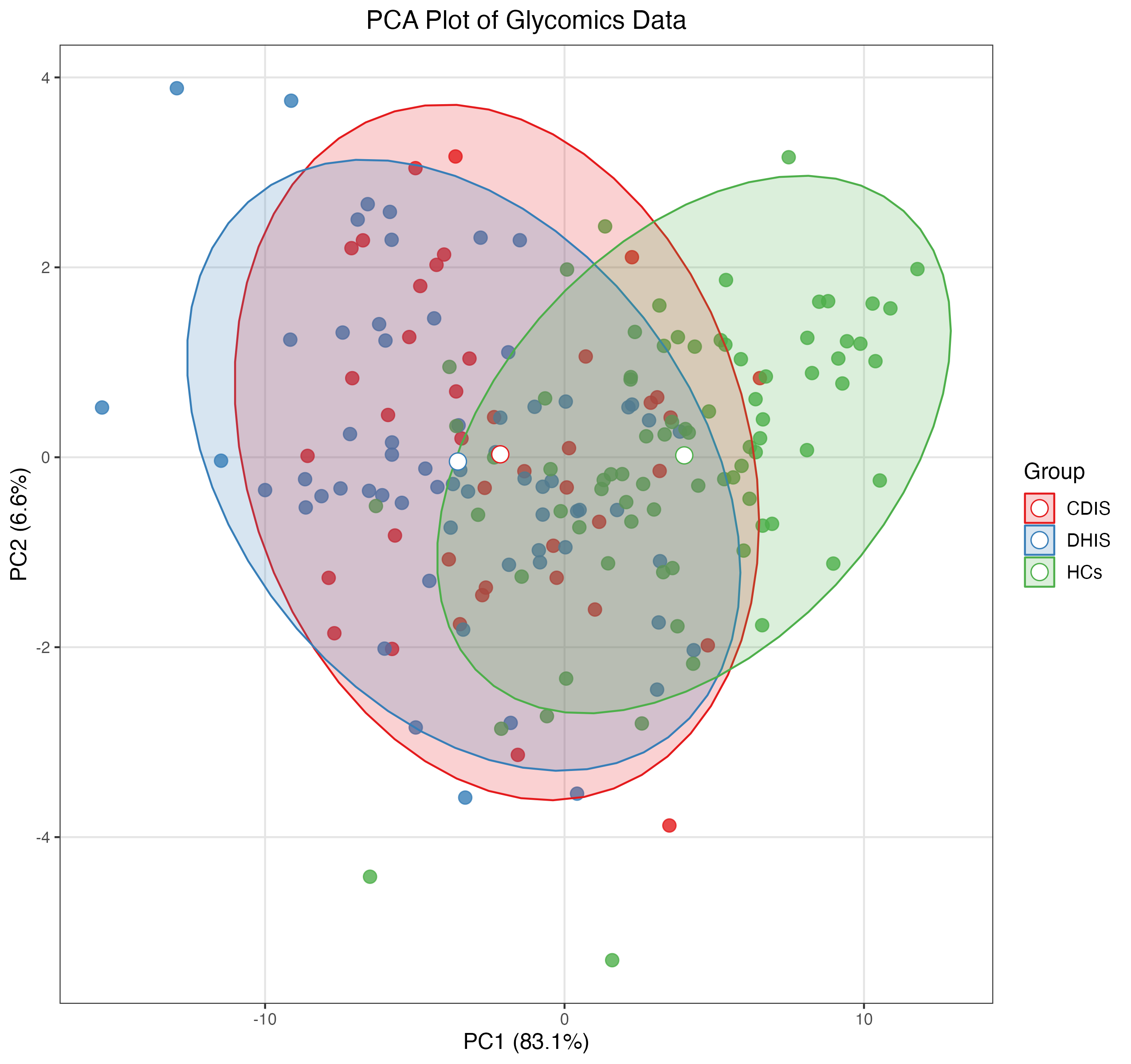


**Supplementary Figure 1. Principal Component Analysis of N-glycan profiles in RA subtypes and healthy controls.** PCA score plot showing the distribution of CDIS (red), DHIS (blue), and HCs (green) with 95% confidence ellipses. The first two principal components explain 89.7% of total variance. CDIS: Cold-dampness impeding syndrome; DHIS: Dampness-heat impeding syndrome; HCs: Healthy Controls.
