# Supplementary material for: Serum IgG N-glycans act as serum biomarkers for differentiation of cold and heat pattern in rheumatoid arthritis
Source: Chin Med. 2025 Nov 6;20:184. doi: 10.1186/s13020-025-01246-3 (PMC12590853; doi:10.1186/s13020-025-01246-3)
Supplement: Supplementary file 2 — Supplementary Material 2 [file 13020_2025_1246_MOESM2_ESM.doc]

**Supplementary Table 1. Traditional Chinese Medicine (TCM) Syndrome Diagnostic Criteria for Rheumatoid Arthritis in 2017**

| **TCM Syndrome** | **Primary Symptom** | **Secondary Symptom** | **Tongue** | **Pulse** |
| --- | --- | --- | --- | --- |
| Cold-dampness impeding syndrome (Cold pattern) | ① Joint pain with a cold sensation, cool to the touch, and without local redness.  ② Pain is aggravated by cold exposure and alleviated by warmth. | ① Joint stiffness and limited movement.  ② Cold limbs, aversion to cold, preference for warmth.  ③ Bland taste in the mouth with no thirst. | Pale and swollen/fat tongue body with a white or greasy coating. | Stringy or Tight |
| Dampness-heat impeding syndrome (Heat pattern) | ① Swollen, hot, and painful joints.  ② Joints feel warm or hot, either subjectively or upon palpation. | ① Local skin redness over the joints.  ② Fever.  ③ Irritability or vexation.  ④ Thirst (or thirst with no desire to drink).  ⑤ Yellow urine. | Red tongue body with a yellow and greasy or thick coating. | Stringy and Slippery or Slippery and Rapid |
